# Supplementary material for: Natural language processing to identify lupus nephritis phenotype in electronic health records
Source: BMC Med Inform Decis Mak. 2024 Mar 3;22(Suppl 2):348. doi: 10.1186/s12911-024-02420-7 (PMC10910523; doi:10.1186/s12911-024-02420-7)
Supplement: Supplementary file 1 — Additional file 1: Table S1. ICD-9/10 codes and LOINC codes used for baseline algorithm. Table S2. Regex concepts and their associated searching keywords for lupus nephritis. Table S3. CUIs and their definition. [file 12911_2024_2420_MOESM1_ESM.docx]

**Additional file table 1S. ICD-9/10 codes and LOINC codes used for baseline algorithm.**

| **Type** | **Terminology** | **Code** | **Note** |
| --- | --- | --- | --- |
| Diagnosis | ICD-9 | 593.6 |  |
| Diagnosis | ICD-9 | 593.81 |  |
| Diagnosis | ICD-9 | 791.7 |  |
| Diagnosis | ICD-10 | N28.0 |  |
| Diagnosis | ICD-10 | N28.9 |  |
| Diagnosis | ICD-10 | R80 | This is a top-level code that contains a tree of other codes. R80 is not used for diagnosis |
| Diagnosis | ICD-10 | R80.9 |  |
| Diagnosis | ICD-10 | R82.99 | This is a top-level code that contains a tree of other codes. R82.99 is not used for diagnosis |
| Laboratory | LOINC | 2889-4 | (value >500mg (/24H), >0.5g (/24H) |
| Laboratory | LOINC | 21482-5 | (value >500mg (/24H), >0.5g (/24H) |
| Laboratory | LOINC | 51790-4 | value >0/hpf or /lpf |
| Laboratory | LOINC | 33804-6 | value >0/hpf or /lpf |
| Laboratory | LOINC | 5807-3 | value >0/hpf or /lpf |

**Abbreviations**: LOINC, logical observation identifiers names and codes; hpf, high-pass filter; lpf, low-pass filter. In the baseline model, a patient is predicted as positive for lupus nephritis if he/she has any positive mention of the ICD or LOINC codes listed in the table above.

**Additional file table 2S. Regex concepts and their associated searching keywords for lupus nephritis**

| **Concepts** | **keywords** |
| --- | --- |
| Proteinuria/red cell cast (renal disorder) | Proteinuria > 0.5 mg |
|  | Urine/creatinine ratio > 0.5 mg/mg |
|  | 24-hour urine protein > 0.5gm |
|  | Red cell cast |
| Nephritis class II | Nephritis class II |
| Nephritis class III | Nephritis class III |
| Nephritis class IV | Nephritis class IV, mesangial proliferative GN |
| Nephritis class V | Nephritis class V, membranous nephritis |

**Additional file table 3S. CUIs and their definition**

| CUIs | Definition |
| --- | --- |
| C0024143 | Glomerulonephritis in the context of systemic lupus erythematosus. |
| C0268757 | Lupus nephritis - WHO Class IV |
| C0268758 | Lupus nephritis - WHO Class V |
| C4053955 | Systemic lupus erythematosus nephritis, with active or inactive diffuse, segmental or global endo- or extracapillary glomerulonephritis involving greater than or equal to 50% of all glomeruli, typically with diffuse subendothelial immune deposits, with or without mesangial alterations. |
| C4053958 | Systemic lupus erythematosus nephritis exhibiting mesangial hypercellularity or mesangial expansion by light microscopy, with mesangial immune deposits. Isolated subepithelial or subendothelial deposits may be visible by immunofluorescence or electron microscopy, but not by light microscopy |
| C4053959 | Systemic lupus erythematosus nephritis with active of inactive focal, segmental or global endo- or extracapillary glomerulonephritis involving less than 50% of all glomeruli, typically with focal subendothelial immune deposits with or without mesangial alterations. |
| C4054543 | Membranous nephritis associated with systemic lupus erythematosus. |
